# Biopolymer-Conjugated Human C-Peptide Provides Sustained Neuroprotection and Preserves Axonal Transport in a Mouse Model of NMDA-Induced Retinal Degeneration via Antioxidative Mechanisms

**DOI:** 10.3390/antiox15070911

**Published:** 2026-07-22

**Authors:** Ji-Seok Yoon, Chan-Hee Moon, Tae-Yong Koh, Woo Ri Cho, Juha Lee, Minsoo Kim, Kwon-Soo Ha

**Affiliations:** 1 Department of Molecular and Cellular Biochemistry, Kangwon National University School of Medicine, Chuncheon 24341, Kangwon-do, Republic of Korea; 2 Biomedical Research Institute, Kangwon National University Hospital, Chuncheon 24289, Kangwon-do, Republic of Korea; 3 Department of Opthalmology, Kangwon National University School of Medicine, Chuncheon 24341, Kangwon-do, Republic of Korea; 4 Department of Anesthesiology, Kangwon National University School of Medicine, Chuncheon 24341, Kangwon-do, Republic of Korea

**Keywords:** glutamate excitotoxicity, K9-C-peptide, NMDA, oxidative stress, retinal neurodegeneration, axonal transport

## Abstract

Glutamate excitotoxicity is a key contributor to the pathogenesis of glaucoma, a leading cause of irreversible blindness worldwide; however, the molecular events driving progressive retinal ganglion cell (RGC) loss and axonal degeneration remain incompletely understood, and effective neuroprotective therapies are lacking. Here, we evaluated the preventive potential of K9-C-peptide, a biopolymer-conjugated human C-peptide, in a mouse model of N-methyl-D-aspartate (NMDA)-induced retinal neurodegeneration and optic nerve axonal transport impairment, and examined potential mechanisms underlying its protective effects. In NMDA-induced excitotoxic mouse retinas, intracellular Ca^2+^ elevation mediated NMDA-induced oxidative stress, including both intracellular and mitochondrial reactive oxygen species (ROS) generation and lipid peroxidation. NMDA exposure induced activation of Müller glia and microglia and upregulation of inflammatory cytokines, ultimately leading to RGC death; these effects were attenuated by prolonged intraocular delivery of ROS scavengers. K9-C-peptide significantly reduced NMDA-induced retinal degeneration, including RGC loss and retinal thinning, and preserved optic nerve axonal transport function in both whole-mount retinas and optic nerve longitudinal sections. These protective effects were associated with suppression of NMDA-induced oxidative stress, mitochondrial dysfunction, and inflammation and reactive gliosis, without altering intracellular Ca^2+^ levels. Notably, sustained intraocular delivery of human C-peptide conferred robust neuroprotection for at least 3 weeks against NMDA-induced retinal degeneration and optic nerve axonal transport impairment. These findings suggest that K9-C-peptide acts as a long-acting neuroprotective agent that mitigates oxidative stress-driven retinal damage and axonal dysfunction, highlighting its translational potential as a C-peptide-based neuroprotective strategy for retinal glutamate excitotoxicity.

## 1. Introduction

Glaucoma is a leading cause of irreversible blindness worldwide [1], characterized by the progressive loss of retinal ganglion cells (RGCs) and degeneration of their axons, ultimately resulting in visual field defects [2]. Elevated intraocular pressure (IOP) is a major risk factor [3], as it induces mechanical stress on RGCs and contributes to disease onset and progression. Accordingly, current glaucoma management primarily relies on IOP-lowering therapies [4,5,6]. However, a substantial proportion of patients continue to exhibit visual field deterioration despite having normal IOP, indicating that IOP-independent mechanisms, such as glutamate excitotoxicity, also play a critical role in glaucoma pathogenesis [4,7,8]. Therefore, elucidating the pathological mechanisms underlying RGC death and identifying effective interventional strategies to prevent RGC degeneration are essential for the management of glaucoma.

Glutamate is the principal excitatory neurotransmitter in the central nervous system, including the retina, where it mediates synaptic transmission of visual signals between neuronal cells [2,6]. In the retina, excessive glutamate induces excitotoxicity primarily through activation of N-methyl-D-aspartate (NMDA) receptors, the predominant glutamate receptors in this tissue [9,10]. Glutamate homeostasis is tightly regulated by its uptake via glutamate transporters, followed by enzymatic conversion into non-toxic glutamine by glutamine synthetase [10,11,12]. Therefore, disruption of this regulatory system—particularly dysfunction of glutamate transporters such as the glutamate-aspartate transporter (GLAST) and reduced glutamine synthetase activity—leads to the accumulation of extracellular glutamate [10,11,13]. The molecular mechanisms underlying glutamate excitotoxicity include intracellular Ca^2+^ overload, oxidative stress, and mitochondrial dysfunction, ultimately resulting in apoptotic death of RGCs and anterograde axonal degeneration [2,9,14]. In addition, reactive gliosis and neuroinflammatory responses further contribute to excitotoxic damage [4,15]. Despite these advances, the precise molecular events that initiate glutamate excitotoxicity and drive subsequent RGC degeneration remain incompletely understood.

Glaucoma treatment primarily focuses on lowering elevated IOP to prevent further damage to the optic nerve [1]. Because IOP is determined by the balance between aqueous humor production and its outflow, current pharmacological strategies are designed either to reduce aqueous humor production or to enhance its drainage [16]. Agents that decrease aqueous humor production include β-blockers, α-adrenergic agonists, and carbonic anhydrase inhibitors, whereas those that increase outflow include prostaglandin analogs and Rho kinase inhibitors [1,16]. Despite their effectiveness in lowering IOP, these therapies do not directly target the underlying neurodegenerative processes affecting RGCs and the optic nerve [1,2,16]. Furthermore, their long-term use is frequently associated with undesirable ocular and systemic side effects, including eye irritation, infection, allergic reactions, and, in rare cases, systemic complications such as organ dysfunction [1,16,17]. Importantly, a substantial proportion of patients continue to experience progressive vision loss despite adequate IOP control, underscoring the limitations of pressure-centered strategies and the need for IOP-independent therapeutic approaches [1,7,16]. Therefore, the development of novel treatments that provide direct neuroprotection, target early pathogenic mechanisms such as excitotoxicity and mitochondrial dysfunction, and ensure sustained intraocular efficacy with minimal side effects remains a critical unmet need.

Proinsulin C-peptide is a 31-amino acid peptide that is co-secreted with insulin from pancreatic β-cells into the bloodstream in equimolar amounts [18,19]. Although initially considered biologically inert due to its lack of effects on glucose and lipid metabolism, C-peptide is now recognized as a bioactive peptide that exerts cytoprotective, vasculoprotective, and neuroprotective effects [20,21,22]. Accumulating evidence indicates that C-peptide exerts protective effects against diabetic complications, including diabetic retinopathy, neuropathy, pulmonary disease, impaired wound healing and cardiovascular disease [20,23,24,25]. These protective effects are associated, at least in part, with reduced oxidative stress through decreased reactive oxygen species (ROS) generation, preserving mitochondrial function, inhibiting transglutaminase 2 activation, and activating AMP-activated protein kinase signaling pathways [26,27].

However, the clinical utility of native C-peptide is constrained by its relatively short plasma half-life (approximately 30 min) [18]. To overcome this limitation, strategies such as covalent conjugation to branched polyethylene glycol and fusion to thermosensitive elastin-like polypeptides (ELPs) have been developed to enhance stability and enable sustained delivery [28,29,30]. In particular, fusion of human C-peptide to a lysine-containing ELP (K9-C-peptide) allows prolonged intraocular or systemic availability and has demonstrated protective effects in diabetic microvascular and macrovascular complications, including diabetic retinopathy, nephropathy, pulmonary diseases, and aortic dysfunction [31,32]. However, whether sustained C-peptide delivery confers an interventional effect against glutamate-induced excitotoxicity remains unknown, and the mechanisms by which C-peptide regulates oxidative and inflammatory pathways underlying RGC death and axonal transport impairment have yet to be elucidated.

In this study, we evaluated the protective efficacy of K9-C-peptide against glutamate excitotoxicity in an NMDA-induced excitotoxicity mouse model. We hypothesized that sustained intraocular release of human C-peptide from K9-C-peptide suppresses oxidative stress-mediated molecular events and confers prolonged retinal neuroprotection. Our findings demonstrate that a single intravitreal injection of K9-C-peptide markedly prevents NMDA-induced RGC death and axonal transport impairment for at least three weeks. These protective effects are associated with suppression of oxidative stress-driven reactive gliosis and inflammation. Collectively, our results identify K9-C-peptide as a long-acting preventive candidate targeting RGC death and axonal transport deficits, and provide insight into the potential mechanisms underlying C-peptide-based interventions in retinal glutamate excitotoxicity.

## 2. Materials and Methods

### 2.1. NMDA-Induced Retinal Excitotoxic Mouse Model

Eight-week-old male C57BL/6 mice were obtained from DBL (Eumseong, Republic of Korea) and maintained under specific pathogen-free conditions in temperature-controlled rooms (22 °C) with a 12 h light/dark cycle, with six mice housed per individually ventilated cage. Mice were provided ad libitum access to standard laboratory chow and water and allowed to acclimatize for at least 4 days prior to the experiments. Following acclimatization, the animals were randomly allocated to the control or NMDA-treated group.

Acute retinal degeneration was induced by a single intravitreal injection of 2 µL of NMDA (10 mmol/L; MilliporeSigma, Burlington, MA, USA) dissolved in PBS using a sterile 10 µL Hamilton syringe (Hamilton, Bonaduz, Switzerland) fitted with a 34-gauge needle. Control mice received an equivalent volume of PBS.

All experimental procedures were conducted in accordance with the guidelines of the National Institutes of Health Guide for the Care and Use of Laboratory Animals and were approved by the Institutional Animal Care and Use Committee of Kangwon National University (approval no. KW-251215-2).

### 2.2. Intravitreal Injections

To optimize the NMDA-induced retinal excitotoxicity model, mice were anesthetized using 3% isoflurane, followed by intravitreal administration of 2 μL of PBS or NMDA at the indicated concentrations (*n* = 3 mice per group). Retinal tissues were analyzed 3 days after injection. In a separate experiment, mice received intravitreal injections of 2 μL of PBS or 10 mmol/L NMDA and were maintained for up to 5 days.

To investigate the effects of pharmacological inhibitors on NMDA-induced retinal oxidative stress, inflammation, reactive gliosis, and apoptosis, mice received an intravitreal injection of 2 µL of PBS, 0.5% thermo-sensitive hydrogel (EDmicBio, Seoul, Republic of Korea), 5 µmol/L BAPTA-AM (Calbiochem, San Diego, CA, USA) in PBS or 0.5% hydrogel (H-BAPTA-AM), 500 mmol/L N-acetyl-L-cysteine (Sigma-Aldrich, St. Louis, MO, USA; NAC) in PBS or 0.5% hydrogel (H-NAC), or 2 µmol/L Trolox (Sigma-Aldrich) in PBS or 0.5% hydrogel (H-Trolox) (*n* = 8 mice per group for the 0.5% hydrogel-treated groups and *n* = 3 mice per group for the PBS-treated groups). One day after treatment, mice received a single intravitreal injection of NMDA and were maintained for 3 days.

To assess the protective effects of human C-peptide on NMDA-induced retinal neurodegeneration and axonal transport dysfunction, mice received an intravitreal injection of 2 μL of PBS, human C-peptide (Peptron, Daejon, Republic of Korea; 0.6 mg/mL; 1.2 μg per eye), K8 polypeptide (8.3 mg/mL; 16.6 μg per eye), or K9–C-peptide (10 mg/mL; 20 μg per eye) (*n* = 21 mice per group), with the doses selected based on our previous studies [26,31]. One day later, mice were given a single intravitreal injection of NMDA and maintained for either 3 days or 3 weeks before tissue collection. Sample sizes were determined based on prior studies using a similar experimental design [26].

### 2.3. Conjugation of K9-C-Peptide and Human C-Peptide with NHS-Fluorescein and Intraocular Fluorescence Imaging

K9-C-peptide, consisting of a human C-peptide—a 31-amino acid peptide released from pancreatic β-cells—fused to a thermosensitive elastin-like polypeptide, and the K8 polypeptide (used as a negative control) were produced by inverse transition cycling, as previously described [31]. Briefly, K9-C-peptide was constructed by genetically linking the gene encoding the K8 polypeptide, which consists of eight repeats of the lysine-containing ELP monomer (ELP-V12K1), to the 5′-end of the gene encoding K1-C-peptide (K1 fused to human C-peptide). The purified K9-C-peptide and K8 polypeptide were characterized by SDS-PAGE.

K9-C-peptide and human C-peptide were labeled with N-hydroxysuccinimide (NHS)-fluorescein (Thermo Fisher Scientific, Waltham, MA, USA). Following intravitreal injection of 2 μL of fluorescein-labeled human C-peptide (0.6 mg/mL; 1.2 μg per eye) or K9-C-peptide (10.0 mg/mL; 20 μg per eye), intraocular fluorescence imaging was performed using confocal microscopy (K1-fluo), as previously described [31].

### 2.4. Immunofluorescence in Retinal Sections and Whole-Mount Retinas

Protein expression in retinal sections or whole-mount retinas from normal or NMDA-treated mice was analyzed by immunofluorescence, as previously described [31]. For retinal section analysis, enucleated eyes were embedded in optimal cutting temperature compound (Sakura Finetek, Torrance, CA, USA), and 10 µm unfixed retinal cryosections were prepared using a microtome–cryostat (Leica Biosystems, Wetzlar, Germany). After fixation with 4% paraformaldehyde for 30 min, cryosections were permeabilized with 0.2% Triton X-100 for 20 min and blocked with 2% bovine serum albumin (BSA) at room temperature for 30 min. For whole-mount analysis, enucleated eyes were fixed with 4% paraformaldehyde for 1 h, and retinas were dissected into a Maltese cross configuration. Retinas were then fixed with acetone for 3 min at −20 °C, permeabilized with 1% Triton X-100 for 4 h, and blocked with 2% BSA overnight at 4 °C. Retina samples were incubated overnight at 4 °C with monoclonal antibodies against tumor necrosis factor-α (TNF-α), interleukin-6 (IL-6), glial fibrillary acidic protein (GFAP), vimentin, or ionized calcium-binding adaptor molecule 1 (Iba-1) (1:200; Cell Signaling Technology, Danvers, MA, USA) or brain-specific homeobox/POU domain protein 3A (Brn3a) (1:200; Abcam, Cambridge, UK). Samples were then incubated with Alexa 546–conjugated goat anti-rabbit IgG (1:200; Invitrogen, Carlsbad, CA, USA) for 2 h. Retinal sections were counterstained with 1 µg/mL Hoechst 33258. Fluorescence images were acquired using a confocal microscope (K1-fluo; Nanoscope Systems, Daejon, Republic of Korea), and protein expression levels were quantified by averaging the fluorescence intensities from three randomly selected microscopic fields per experiment (*n* = 6).

### 2.5. Hematoxylin and Eosin Staining of Mouse Retinal Sections

Hematoxylin and eosin (H&E) staining was carried out, as previously described [18]. Retinal cryosections (10 µm) located approximately 0.5–0.7 mm from the optic nerve head were fixed with Davidson’s fixative for 2 h and cryoprotected overnight in 30% sucrose. Sections were stained with Harris hematoxylin solution (MilliporeSigma) for 10 min, followed by 0.2% eosin for 1 min. Stained sections were imaged using bright-field microscopy.

### 2.6. Measurement of Neuronal Apoptosis in Mouse Retinal Sections

Neuronal apoptosis in mouse retinas was assessed using the APO-BrdU TUNEL assay kit (BD Biosciences, San Jose, CA, USA) as previously described [18]. Retinal cryosections were sequentially fixed with 1% (*w*/*v*) paraformaldehyde for 30 min and 70% (*v*/*v*) ethanol for 30 min on ice, followed by incubation with a DNA-labeling solution containing terminal deoxynucleotidyl transferase and 5-bromo-2′-deoxyuridine for 1 h. Sections were then incubated with an FITC-conjugated anti-BrdU antibody for 30 min and counterstained with 1 µg/mL Hoechst 33258 (MilliporeSigma, Burlington, MA, USA) for 10 min. TUNEL-positive cells were visualized by confocal microscopy (K1-Fluo) and quantified by counting positive cells in three randomly selected fields per section (*n* = 6).

### 2.7. Measurements of Intracellular Ca^2+^ Levels in Mouse Retinal Sections

Intracellular Ca^2+^ levels in retinal cryosections were measured using Fluo-4 AM (Thermo Fisher Scientific). Unfixed retinal cryosections were incubated with 2 µmol/L Fluo-4 AM at 37 °C for 30 min. Fluorescence images were acquired using confocal microscopy, and intracellular Ca^2+^ levels were quantified by averaging the fluorescence intensities from three randomly selected microscopic fields per experiment (*n* = 6).

### 2.8. Measurements of Intracellular and Mitochondrial ROS and Mitochondrial Membrane Potential (∆Ψm) in Mouse Retinal Sections

Intracellular ROS and superoxide levels in retinal sections were measured using CellROX™ Green (Thermo Fisher Scientific) and dihydroethidium (DHE; Thermo Fisher Scientific), respectively, as previously described [18]. Mitochondrial ROS levels were assessed using MitoSOX™ Red (Thermo Fisher Scientific) [33]. Unfixed retinal cryosections were incubated with 5 µmol/L CellROX™ Green, 5 µmol/L DHE, or 5 µmol/L MitoSOX™ Red at 37 °C for 30 min. Fluorescence images were acquired using confocal microscopy, and intracellular and mitochondrial ROS levels were quantified by averaging the fluorescence intensities from three randomly selected microscopic fields per experiment (*n* = 6).

Mitochondrial membrane potential (∆Ψm) was determined using 2 µmol/L JC-1 dye (Thermo Fisher Scientific) and confocal microscopy [34]. Data are expressed as the ratio of J-aggregates (red) to monomers (green) fluorescence intensity (*n* = 6).

### 2.9. Measurement of Lipid Peroxidation by MDA Assay in Mouse Retinas

Malondialdehyde (MDA) levels, an indicator of lipid peroxidation, in mouse retinas were measured using a commercially available MDA assay kit (Sigma-Aldrich). Retinal lysates (100 μL) were mixed with thiobarbituric acid solution (300 μL), incubated at 95 °C for 60 min, and centrifuged at 13,000× *g* for 10 min. MDA concentrations were determined according to the manufacturer’s instructions (*n* = 5).

### 2.10. Measurement of Inflammatory Cytokine Levels by ELISA in Mouse Retinas

TNF-α and IL-6 levels in mouse retinas were determined using commercially available ELISA kits for TNF-α (Abcam) and IL-6 (Invitrogen). Protein extracts were prepared from retinas (two retinas per treatment) using lysis buffer and centrifuged at 17,600× *g* for 15 min at 4 °C [27]. Cytokine concentrations were measured according to the manufacturers’ instructions and quantified using a microplate spectrophotometer (Epoch; BioTek, Winooski, VT, USA; *n* = 5).

### 2.11. Measurement of Retinal Thickness and Degeneration by Optical Coherence Tomography

Retinal thickness and degeneration were evaluated by optical coherence tomography (OCT). For image acquisition, mice were anesthetized with 3% isoflurane, and pupils were dilated using a single drop of Mydrin-P (Santen Pharmaceutical, Osaka, Japan). To prevent corneal dehydration during the procedure, ophthalmic gel (Samil Pharmaceutical, Seoul, Republic of Korea) was applied to the ocular surface before imaging. OCT images were captured using a Micron IV retinal imaging system (Phoenix MICRON, Bend, OR, USA) equipped with an ultrabroadband superluminescent diode light source centered at 830 nm with a bandwidth of 160 nm, providing a retinal imaging field of 1.8 mm with an axial pixel resolution of 1.8 μm. Total retinal thickness (extending from the inner limiting membrane to Bruch’s membrane) and inner retinal layer (IRL) thicknesses were measured at a fixed distance of approximately 300 µm from the optic nerve head using the integrated analysis software (*n* = 6).

### 2.12. Assessment of Axonal Transport Using Cholera Toxin Subunit B

Anterograde axonal transport was assessed by intravitreal administration of Alexa Fluor 488-conjugated cholera toxin subunit B (CTB; Invitrogen). Under 3% isoflurane anesthesia, mice received a 2 µL intravitreal injection of CTB solution (0.5 mg/mL). For whole-mount retinal analysis, enucleated eyes were fixed with 4% paraformaldehyde, and retinas were dissected into a Maltese cross configuration. For optic nerve analysis, optic nerves were fixed with 4% paraformaldehyde, embedded in optimal cutting temperature compound, and cryosectioned longitudinally at a thickness of 10 µm using a microtome–cryostat. Fluorescence images were acquired using confocal microscopy, and CTB levels were quantified by averaging the fluorescence intensities from three randomly selected microscopic fields per experiment (*n* = 6).

### 2.13. Statistical Analysis

Statistical analyses were performed using OriginPro 2015 (OriginLab, Northampton, MA, USA) and are expressed as the mean ± standard deviation (SD). The sample size was *n* = 6 per group for imaging studies and *n* = 5 per group for Western blot or ELISA analyses. Statistical significance was determined using one-way analysis of variance (ANOVA) with Holm–Sidak’s multiple comparisons test. Differences were considered statistically significant at *p* < 0.05.

## 3. Results

### 3.1. K9-C-Peptide Attenuates RGC Death in an NMDA-Induced Excitotoxic Mouse Model

**Figure 1:**
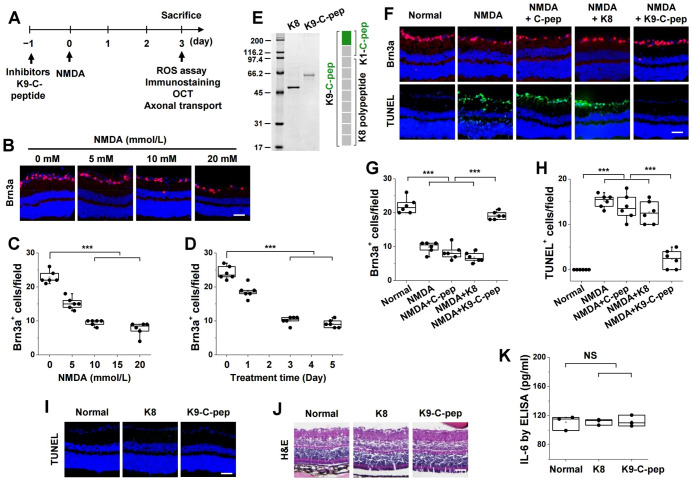
**K9-C-peptide is non-toxic to the retina and attenuates RGC death in an NMDA-induced excitotoxic mouse model.** (**A**) Schematic illustration of the NMDA-induced excitotoxic mouse model and experimental design. (**B**–**D**) C57BL/6 mice were intravitreally injected with NMDA (5–20 mmol/L) and analyzed after 3 days (**B**,**C**), or injected with NMDA (10 mmol/L) and analyzed at 1–5 days (**D**), followed by Brn3a immunofluorescence analysis. (**B**) Representative images of Brn3a expression (red) with Hoechst 33258 nuclear counterstaining (blue). Scale bar, 50 µm. (**C**,**D**) Quantification of Brn3a^+^ cells (*n* = 6). (**E**) Coomassie staining of purified K8 polypeptide (K8) and K9-C-peptide (K9-C-pep). (**F**–**H**) C57BL/6 mice received intravitreal injections of PBS, human C-peptide (C-pep), K8, or K9-C-pep 1 day prior to intravitreal injection of NMDA (10 mmol/L). Retinas were analyzed 3 days post-injection for retinal ganglion cell (RGC) death using Brn3a immunofluorescence and TUNEL assays. (**F**) Representative images of Brn3a expression (red) and TUNEL-positive cells (green) with Hoechst 33258 nuclear counterstaining (blue). Scale bars, 50 µm. (**G**,**H**) Quantification of Brn3a^+^ cells and TUNEL^+^ cells (*n* = 6). (**I**–**K**) No cytotoxicity was observed in normal mice after intravitreal injection of K8 polypeptide (K8) or K9-C-peptide. (**I**) No apoptosis was detected in retinal sections by TUNEL staining (green). Scale bar, 50 μm. (**J**) H&E staining revealed no histopathological changes in retinal sections. Scale bar, 50 µm. (**K**) ELISA showed no change in interleukin-6 (IL-6) levels in retinal lysates. Statistical significance was determined using one-way ANOVA followed by Holm–Sidak’s multiple comparisons test. *** *p* < 0.001.

To investigate the preventive efficacy of K9-C-peptide against glutamate excitotoxicity in the retina, we first established an optimized NMDA-induced excitotoxic mouse model. Mice received intravitreal injections of NMDA at doses ranging from 5 to 20 mmol/L (2 μL), and retinal RGC degeneration was assessed after 3 days using Brn3a immunofluorescence (Figure 1A). NMDA induced a dose-dependent reduction in Brn3a^+^ cells, with a pronounced effect observed at 10 mmol/L (Figure 1B,C). To determine the optimal exposure duration, RGC degeneration was evaluated at 1–5 days post-injection. Brn3a immunostaining revealed a marked decrease in Brn3a^+^ cell number at 3 days (Figure 1D). These results indicate that intravitreal administration of NMDA (10 mmol/L) with a 3-day post-injection period provides a reproducible model for assessing preventive interventions.

We next examined the protective effect of K9-C-peptide in this excitotoxic model. K9-C-peptide was constructed by genetically linking K1-C-peptide to the K8 polypeptide, whereas the K8 polypeptide alone was used as a negative control. Both recombinant proteins were produced in *E. coli*, purified, and verified by SDS-PAGE (Figure 1E). Pharmacokinetic analysis using intraocular fluorescence imaging of fluorescein-conjugated K9-C-peptide and human C-peptide (administered at an equimolar dose relative to K9-C-peptide) revealed that K9-C-peptide persisted for up to 3 weeks, whereas native human C-peptide became undetectable by 3 days (Figure S1), suggesting prolonged intraocular retention of K9-C-peptide.

Brn3a immunofluorescence demonstrated that K9-C-peptide significantly attenuated NMDA-induced RGC loss (Figure 1F,G). This neuroprotective effect was further supported by TUNEL assays, which showed reduced apoptotic cell death in K9-C-peptide-treated retinas (Figure 1F,H). In contrast, neither an equimolar dose of human C-peptide to K9-C-peptide nor the K8 polypeptide conferred protection against NMDA-induced excitotoxicity, indicating that sustained delivery is required for the observed protective effect of K9-C-peptide. Importantly, intravitreal administration of K8 polypeptide or K9-C-peptide did not induce RGC apoptosis, retinal structural abnormalities, or inflammatory responses in normal mice (Figure 1I–K).

### 3.2. Intracellular Ca^2+^ Elevation Mediates NMDA-Induced Oxidative Stress in Excitotoxic Mouse Retinas

**Figure 2:**
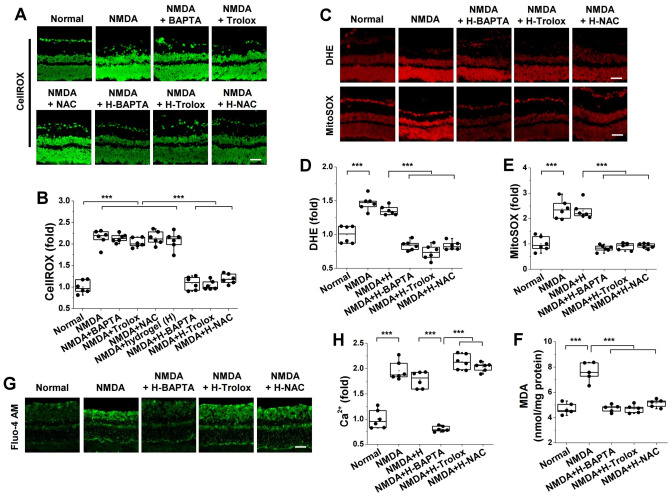
**Intracellular Ca^2+^ elevation mediates NMDA-induced oxidative stress in excitotoxic mouse retinas.** Mice received intravitreal injections of BAPTA-AM (BAPTA), Trolox, NAC, hydrogel (H; 0.5%), BAPTA-AM in hydrogel (H-BAPTA), Trolox in hydrogel (H-Trolox), or NAC in hydrogel (H-NAC) 1 day prior to intravitreal injection of NMDA (10 mmol/L). Retinas were analyzed 3 days post-injection for intracellular ROS, mitochondrial ROS, superoxide generation, intracellular Ca^2+^ levels, and lipid peroxidation. (**A**,**B**) Intracellular ROS generation was visualized using CellROX™ Green (**A**) and quantified by fluorescence intensity (**B**; *n* = 6). Scale bar, 50 µm. (**C**–**E**) Superoxide and mitochondrial ROS generation were assessed using dihydroethidium (DHE) and MitoSOX™ Red, respectively. (**C**) Representative fluorescence images. Scale bars, 50 µm. (**D**,**E**) Quantification of superoxide (**D**) and mitochondrial ROS (**E**) levels (*n* = 6). (**F**) Quantification of malondialdehyde (MDA) levels (*n* = 5). (**G**,**H**) Intracellular Ca^2+^ levels were visualized using Fluo-4 AM (**G**) and quantified by fluorescence intensity (**H**; *n* = 6). Scale bar, 50 µm. Statistical significance was determined using one-way ANOVA followed by Holm–Sidak’s multiple comparisons test. *** *p* < 0.001.

To explore the molecular mechanism underlying NMDA-induced RGC death, we assessed oxidative stress in excitotoxic mouse retinas. NMDA markedly increased intracellular ROS generation, which was suppressed by intravitreal injection of H-Trolox or H-NAC (Figure 2A,B). In contrast, Trolox or NAC administered alone did not attenuate ROS levels, suggesting that hydrogel formulation enables sustained intraocular delivery of ROS scavengers.

H-BAPTA-AM also significantly inhibited NMDA-induced ROS generation, whereas BAPTA-AM alone had no effect, supporting an upstream role for intracellular Ca^2+^ elevation in ROS production (Figure 2A,B). This was further validated using DHE and MitoSOX™, which showed that NMDA markedly increased superoxide production and mitochondrial ROS generation; both were suppressed by H-BAPTA-AM, as well as by H-Trolox and H-NAC (Figure 2C–E). Consistently, NMDA-induced lipid peroxidation, assessed by malondialdehyde levels, was significantly attenuated by H-BAPTA-AM, H-Trolox, or H-NAC (Figure 2F). Notably, NMDA elevated intracellular Ca^2+^ levels, and this increase was inhibited by H-BAPTA-AM but not by H-Trolox or H-NAC (Figure 2G,H).

### 3.3. Inhibition of ROS Generation Suppresses NMDA-Induced Reactive Gliosis, Inflammatory Cytokine Expression, and RGC Loss in Excitotoxic Mouse Retinas

**Figure 3:**
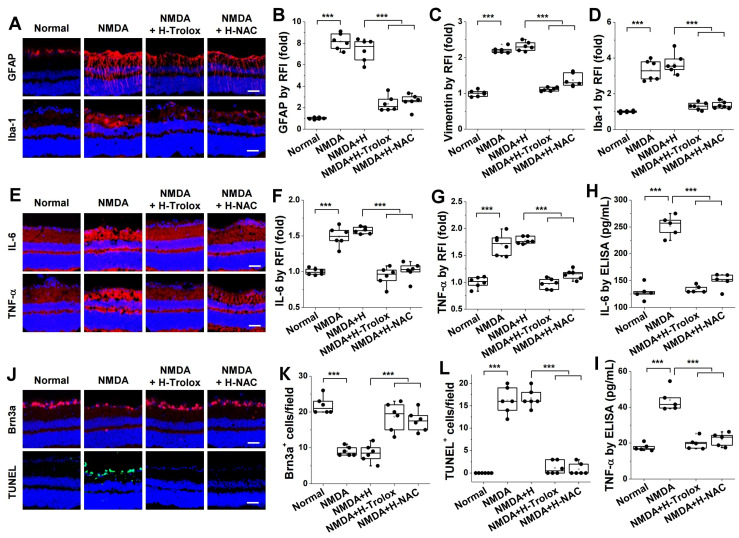
Inhibition of ROS generation suppresses NMDA-induced reactive gliosis, inflammatory cytokine expression, and RGC death in excitotoxic mouse retinas. Mice received intravitreal injections of hydrogel, H-Trolox, or H-NAC 1 day prior to intravitreal injection of NMDA (10 mmol/L). Retinas were analyzed 3 days post-injection for reactive gliosis, inflammatory cytokine expression, and RGC death using immunofluorescence and ELISA. (**A**–**D**) Reactive gliosis was evaluated by immunofluorescence analysis of glial fibrillary acidic protein (GFAP), vimentin, and ionized calcium-binding adaptor molecule 1 (Iba-1). (**A**) Representative fluorescence images of GFAP and Iba-1 expression (red) with Hoechst 33258 nuclear counterstaining (blue). Scale bars, 50 µm. (**B**–**D**) Quantification of GFAP (**B**), vimentin (**C**), and Iba-1 (**D**) expression based on the relative fluorescence intensity (RFI; *n* = 6). (**E**–**I**) Inflammatory cytokine expression was assessed by immunofluorescence and ELISA. (**E**) Representative fluorescence images of IL-6 and TNF-α expression (red) with Hoechst 33258 nuclear counterstaining (blue). Scale bars, 50 µm. (**F**,**G**) Quantification of IL-1β (**F**) and TNF-α (**G**) expression by RFI (*n* = 6). (**H**,**I**) Quantification of IL-1β (**H**) and TNF-α (**I**) levels by ELISA (*n* = 5). (**J**–**L**) RGC death was evaluated using Brn3a immunofluorescence staining and TUNEL assays. (**J**) Representative fluorescence images of Brn3a expression (red) and TUNEL-positive cells (green) with Hoechst 33258 nuclear counterstaining (blue). Scale bars, 50 µm. (**K**,**L**) Quantification (*n* = 6) of Brn3a^+^ cells and TUNEL^+^ cells. Statistical significance was determined using one-way ANOVA followed by Holm–Sidak’s multiple comparisons test. *** *p* < 0.001.

We investigated the role of ROS generation in NMDA-induced reactive gliosis, inflammatory cytokine expression, and RGC loss in excitotoxic mouse retinas. Reactive gliosis was assessed by Müller glial activation using immunofluorescence staining for GFAP and vimentin, and by microglial activation using Iba-1 staining. NMDA increased the expression of GFAP and vimentin, which was markedly suppressed by H-Trolox or H-NAC (Figure 3A–C). Consistently, NMDA-induced microglial activation was also attenuated by H-Trolox or H-NAC (Figure 3A,D), whereas hydrogel alone had no effect. These findings indicate a critical role of ROS generation in NMDA-induced reactive gliosis.

We next examined the involvement of ROS generation in NMDA-induced inflammatory cytokine expression. Immunofluorescence staining showed that NMDA-induced expression of IL-6 and TNF-α was significantly reduced by H-Trolox or H-NAC (Figure 3E–G). In contrast, hydrogel alone had no effect. Consistent results were obtained by ELISA (Figure 3H,I), further supporting a key role of ROS generation in NMDA-induced inflammation.

Finally, we assessed the role of ROS generation in NMDA-induced RGC loss using Brn3a immunofluorescence and TUNEL assays. Sustained intraocular delivery of ROS scavengers prevented NMDA-induced RGC loss, whereas hydrogel alone showed no protective effect (Figure 3J–L).

### 3.4. K9-C-Peptide Inhibits NMDA-Induced Oxidative Stress, Mitochondrial Dysfunction, Reactive Gliosis, and Inflammatory Cytokine Expression

**Figure 4:**
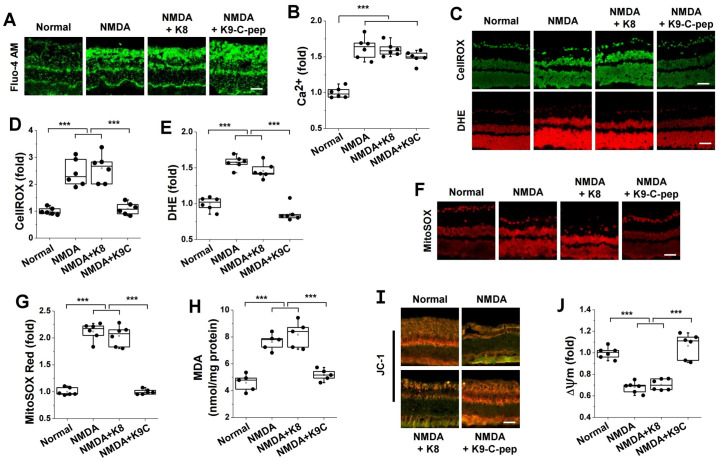
K9-C-peptide inhibits NMDA-induced oxidative stress and mitochondrial membrane potential collapse in excitotoxic mouse retinas. Mice received intravitreal injections of K8 or K9-C-pep 1 day prior to intravitreal injection of NMDA (10 mmol/L). Retinas were analyzed 3 days post-injection for intracellular Ca^2+^ levels, oxidative stress, and mitochondrial membrane potential (∆Ψm). (**A**,**B**) Intracellular Ca^2+^ levels were visualized using Fluo-4 AM (**A**) and quantified by fluorescence intensity ((**B**); *n* = 6). Scale bar, 50 µm. (**C**–**G**) Oxidative stress was assessed by measuring intracellular ROS and superoxide generation, mitochondrial ROS generation, and lipid peroxidation. (**C**–**E**) Intracellular ROS and superoxide generation were evaluated using CellROX™ Green and DHE, respectively. (**C**) Representative fluorescence images. Scale bars, 50 µm. (**D**,**E**) Quantification of intracellular ROS (**D**) and superoxide (**E**) generation (*n* = 6). (**F**,**G**) Mitochondrial ROS generation was visualized using MitoSOX™ Red (**F**) and quantified by fluorescence intensity (**G**; *n* = 6). Scale bar, 50 µm. (**H**) Quantification of MDA levels (*n* = 5). (**I**,**J**) ∆Ψm was visualized using JC-1 (**I**) and quantified as the ratio of J-aggregates (red) to monomers (green) ((**J**); *n* = 6). Scale bar, 50 µm. Statistical significance was determined using one-way ANOVA followed by Holm–Sidak’s multiple comparisons test. *** *p* < 0.001.

To investigate the molecular mechanisms responsible for the preventive effect of K9-C-peptide against NMDA-induced RGC loss, we first evaluated its effects on intracellular Ca^2+^ levels and oxidative stress in excitotoxic mouse retinas. NMDA significantly increased intracellular Ca^2+^ levels; however, this elevation was not affected by K9-C-peptide (Figure 4A,B). In contrast, K9-C-peptide markedly suppressed NMDA-induced ROS and superoxide generation (Figure 4C–E). Consistently, NMDA-induced mitochondrial ROS production and lipid peroxidation were also significantly attenuated by K9-C-peptide (Figure 4F–H).

We next assessed whether K9-C-peptide modulates NMDA-induced mitochondrial dysfunction by measuring ΔΨm. JC-1 staining revealed that NMDA-induced loss of ΔΨm was significantly prevented by K9-C-peptide, whereas K8 had no protective effect (Figure 4I,J).

**Figure 5:**
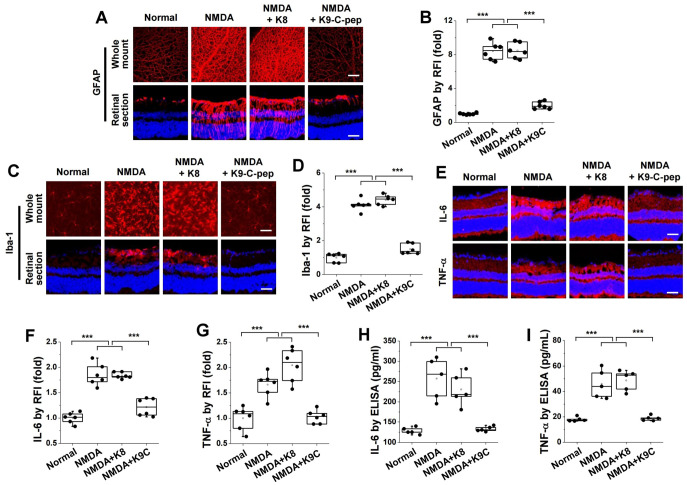
K9-C-peptide attenuates NMDA-induced reactive gliosis and inflammatory cytokine expression in excitotoxic mouse retinas. Mice received intravitreal injections of K8 or K9-C-peptide 1 day prior to intravitreal injection of NMDA (10 mmol/L). Retinas were analyzed 3 days post-injection for markers of gliosis and inflammatory cytokine expression. (**A**–**D**) Reactive gliosis was evaluated by immunofluorescence analysis of GFAP and Iba-1 expression. (**A**,**B**) GFAP expression (red) was visualized with Hoechst 33258 nuclear counterstaining (blue) in whole-mount retinas and retinal sections (**A**) and quantified based on the relative fluorescence intensity (RFI) in retinal sections (*n* = 6) (**B**) Scale bars, 100 µm (whole-mount retinas) and 50 µm (retinal sections). (**C**,**D**) Iba-1 expression (red) was visualized with Hoechst 33258 nuclear counterstaining (blue) using immunofluorescence in whole-mount retinas and retinal sections (**C**) and quantified based on the RFI in retinal sections ((**D**); *n* = 6). Scale bars, 50 µm. (**E**–**I**) Inflammatory cytokine expression was assessed by immunofluorescence and ELISA. (**E**–**G**) IL-6 and TNF-α expression were analyzed by immunofluorescence. (**E**) Representative fluorescence images of IL-6 and TNF-α expression (red) with Hoechst 33258 nuclear counterstaining (blue). Scale bars, 50 µm. (**F**,**G**) Quantification of IL-6 (**F**) and TNF-α (**G**) based on the RFI (*n* = 6). (**H**,**I**) Quantification of IL-6 (**H**) and TNF-α (**I**) levels by ELISA (*n* = 5). Statistical significance was determined using one-way ANOVA followed by Holm–Sidak’s multiple comparisons test. *** *p* < 0.001.

Finally, we examined the protective effects of K9-C-peptide on NMDA-induced reactive gliosis and inflammatory cytokine expression. Reactive gliosis was evaluated by immunofluorescence staining of GFAP and Iba-1. NMDA increased GFAP expression in whole-mount retinas and retinal sections, and this increase was suppressed by K9-C-peptide, whereas K8 had no effect (Figure 5A,B). Consistently, NMDA-induced microglial activation was significantly inhibited by K9-C-peptide, but not by K8 (Figure 5C,D). Moreover, NMDA-induced expression of IL-6 and TNF-α, which was assessed by immunofluorescence and ELISA, was markedly attenuated by K9-C-peptide, but not by K8 (Figure 5E–I).

### 3.5. K9-C-Peptide Ameliorates NMDA-Induced Retinal Degeneration and Axonal Transport Impairment

**Figure 6:**
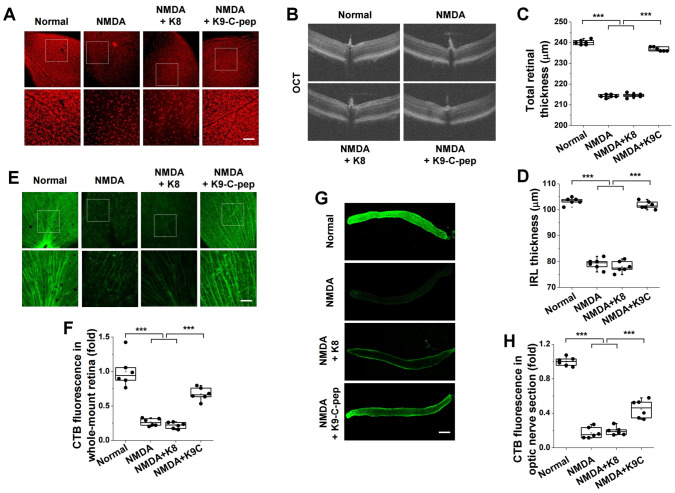
K9-C-peptide attenuates NMDA-induced retinal degeneration and axonal transport impairment in excitotoxic mouse retinas. Mice received intravitreal injections of K8 or K9-C-peptide 1 day prior to intravitreal injection of NMDA (10 mmol/L). Retinas were analyzed 3 days post-injection for RGC loss, retinal thickness, and axonal transport using cholera toxin subunit B (CTB) conjugated to Alexa Fluor 488. (**A**) Brn3a expression was visualized using immunofluorescence in whole-mount retinas. Enlarged images of selected regions are shown below each panel. Scale bar, 100 µm. (**B**–**D**) Total retinal and inner retinal layer (IRL) thicknesses were assessed by optical coherence tomography (OCT) imaging. (**B**) Representative OCT images. (**C**,**D**) Quantification of total retinal and IRL thicknesses (*n* = 6). (**E**–**H**) Axonal transport was assessed using CTB labeling in whole-mount retinas and optic nerve longitudinal sections. (**E**,**F**) Axonal transport was visualized in whole-mount retinas (**E**) and quantified by fluorescence intensity (**F**; *n* = 6). Enlarged images of selected regions are shown below each panel. Scale bar, 100 µm. (**G**,**H**) Axonal transport was visualized in optic nerve longitudinal sections (**G**) and quantified by fluorescence intensity ((**H**); *n* = 6). Scale bar, 1000 µm. Statistical significance was determined using one-way ANOVA followed by Holm–Sidak’s multiple comparisons test. *** *p* < 0.001.

To further investigate the protective effects of K9-C-peptide against NMDA-induced RGC loss, we examined retinal degeneration using Brn3a immunofluorescence staining and OCT in excitotoxic mouse retinas. NMDA significantly decreased the number of Brn3a^+^ cells in whole-mount retinas, and this reduction was significantly reversed by K9-C-peptide, whereas K8 had no effect (Figure 6A). Consistently, total retinal and IRL thicknesses, as assessed by OCT, were significantly decreased by NMDA, and this retinal thinning was attenuated by K9-C-peptide, but not by K8 (Figure 6B,D).

We next evaluated the protective effect of K9-C-peptide against NMDA-induced axonal transport impairment using CTB tracing. NMDA dramatically decreased axonal transport in whole-mount retinas, and this reduction was significantly restored by K9-C-peptide, whereas K8 had no effect (Figure 6E,F). Consistently, in optic nerve longitudinal sections, NMDA-induced axonal transport impairment was significantly ameliorated by K9-C-peptide, but not by K8 (Figure 6G,H).

### 3.6. Long-Term Preventive Effects of K9-C-Peptide

**Figure 7:**
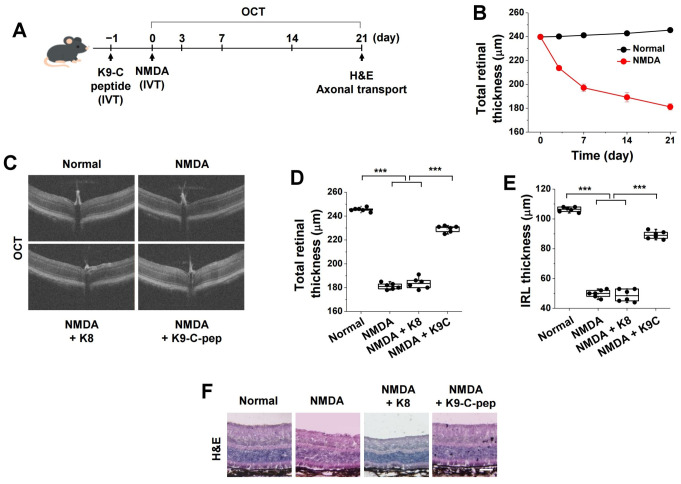
**Long-term neuroprotective effects of K9-C-peptide against NMDA-induced retinal degeneration in mouse retinas.** Mice received intravitreal injections of PBS, K8, or K9-C-peptide 1 day prior to intravitreal injection of NMDA (10 mmol/L) and were monitored for up to 3 weeks post-injection. Retinas were analyzed for retinal thickness and histological changes. (**A**) Schematic illustration of the experimental design. (**B**) NMDA-induced retinal thinning was assessed over a 3 week period using OCT (*n* = 6). (**C**–**E**) Total retinal and IRL thicknesses were assessed by OCT imaging. (**C**) Representative OCT images. (**D**,**E**) Quantification of total retinal and IRL thicknesses (*n* = 6). (**F**) Representative hematoxylin and eosin (H&E)-stained retinal sections. Scale bar, 50 µm. Statistical significance was determined using one-way ANOVA followed by Holm–Sidak’s multiple comparisons test. *** *p* < 0.001.

To evaluate the long-term preventive efficacy of sustained human C-peptide delivery, mice received an intravitreal injection of K9-C-peptide before NMDA administration and were monitored for 3 weeks (Figure 7A). Preventive effects were examined using OCT and H&E staining. OCT imaging revealed that NMDA progressively reduced total retinal thickness over 3 weeks to approximately 180 μm (Figure 7B). Both total retinal and IRL thinning induced by NMDA were significantly attenuated by K9-C-peptide, but not by K8 (Figure 7C–E). Consistent with the OCT findings, H&E staining demonstrated marked inner retinal degeneration, characterized by thinning of the GCL and inner plexiform layer and disruption of inner retinal architecture following NMDA administration (Figure 7F).

**Figure 8:**
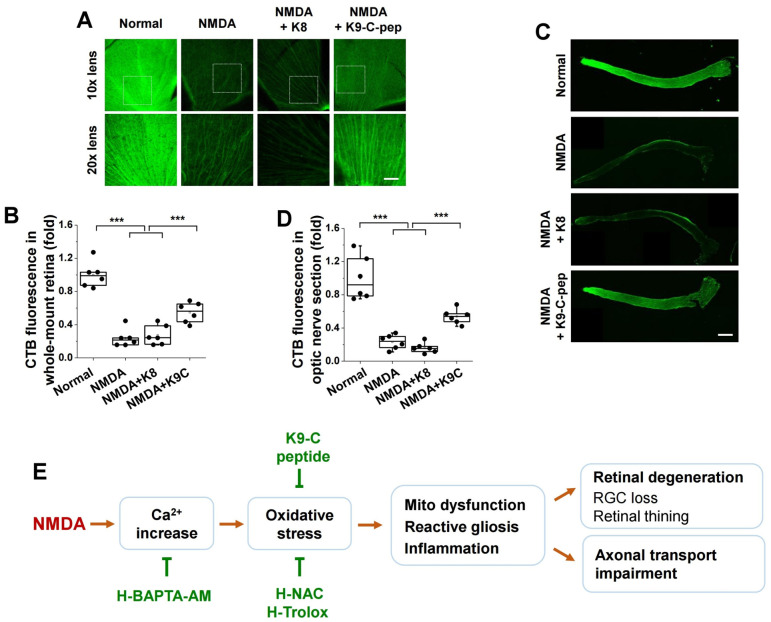
Long-term protective effects of K9-C-peptide against NMDA-induced axonal transport impairment in excitotoxic mouse retinas. Mice received intravitreal injections of PBS, K8, or K9-C-peptide 1 day prior to intravitreal injection of NMDA (10 mmol/L) and were maintained for 3 weeks post-injection. Retinas were analyzed for axonal transport function. (**A**,**B**) Axonal transport was visualized in whole-mount retinas (**A**) and quantified by fluorescence intensity (**B**; *n* = 6). Enlarged images of selected regions are shown below each panel. Scale bar, 100 µm. (**C**,**D**) Axonal transport was visualized in optic nerve longitudinal sections (**C**) and quantified by fluorescence intensity ((**D**); *n* = 6). Scale bar, 1000 µm. (**E**) Proposed mechanism underlying the long-term neuroprotective effects of K9-C-peptide in NMDA-induced retinal excitotoxicity. Statistical significance was determined using one-way ANOVA followed by Holm–Sidak’s multiple comparisons test. Mito dysfunction, mitochondrial dysfunction; *** *p* < 0.001.

We next evaluated the preventive effects of K9-C-peptide on NMDA-induced axonal transport impairment using CTB tracing. NMDA severely decreased axonal transport in whole-mount retinas, and this reduction was significantly restored by K9-C-peptide, whereas K8 had no effect (Figure 8A,B). Consistently, in optic nerve longitudinal sections, NMDA-induced axonal transport impairment was significantly ameliorated by K9-C-peptide, but not by K8 (Figure 8C,D).

## 4. Discussion

In the present study, we identified K9-C-peptide as a long-acting neuroprotective candidate for preventing retinal neurodegeneration and axonal transport impairment in an NMDA-induced excitotoxicity mouse model. Our experimental strategy was based on the rationale that conjugating human C-peptide to a thermosensitive ELP would prolong its intraocular retention and bioavailability, thereby overcoming the short half-life of native C-peptide and enabling sustained preventive activity following a single intravitreal injection. This approach was well aligned with the primary aim of evaluating whether long-term delivery of C-peptide could provide durable neuroprotection against excitotoxic retinal injury. K9-C-peptide protected against NMDA-induced RGC loss and retinal thinning, as well as axonal transport impairment, and these protective effects were associated with the suppression of oxidative stress, mitochondrial dysfunction, reactive gliosis, and inflammation (Figure 8E). Prolonged intraocular delivery of human C-peptide using a single intravitreal injection of K9-C-peptide provided sustained protection against NMDA-induced retinal degeneration and preserved axonal transport function for at least three weeks. These findings demonstrate that ELP conjugation not only enhanced the preventive durability of C-peptide but also represents a practical strategy for improving the translational potential of peptide-based therapies for retinal neurodegenerative diseases. Collectively, our findings suggest that K9-C-peptide is a long-acting neuroprotective candidate against glutamate excitotoxicity.

Glaucoma is a leading cause of irreversible blindness worldwide [1]. Elevated IOP is a major risk factor, and thus current pharmacological strategies have focused on IOP-lowering therapies, which reduce aqueous humor production or enhance its drainage [2,7,16]. Despite these pharmacotherapies being effective in lowering IOP, a substantial proportion of patients continue to exhibit visual field deterioration even at normal IOP, indicating the involvement of IOP-independent mechanisms in glaucoma pathogenesis [2,4]. Moreover, these therapeutic agents, including carbonic anhydrase inhibitors, β-blockers, prostaglandin analogs, and parasympathomimetics, are frequently associated with undesirable ocular and systemic side effects, such as eye irritation, infection, allergic reactions, and, in rare cases, systemic complications including organ dysfunction [1,16,17]. In the retina, glutamate excitotoxicity is a key pathological process in glaucoma, in which excessive glutamate overstimulates NMDA receptors, leading to intracellular Ca^2+^ overload, oxidative stress, and mitochondrial dysfunction, ultimately resulting in RGC death and anterograde axonal transport impairment [2,6,9,35]. Therefore, therapeutic approaches targeting glutamate excitotoxicity–associated retinal degeneration are required for the management of glaucoma [36].

Our findings suggest that, in the retinas of NMDA-induced glutamate excitotoxicity mice, NMDA-induced RGC loss potentially occurs through intracellular Ca^2+^ overload-mediated oxidative stress, including both intracellular and mitochondrial ROS generation and lipid peroxidation, activation of Müller glia and microglia, as well as upregulation of inflammatory cytokines, ultimately leading to RGC death. These pathological events were inhibited by sustained intraocular delivery of ROS scavengers using a thermosensitive hydrogel. K9-C-peptide attenuated NMDA-induced retinal degeneration and axonal transport impairment, potentially through suppression of NMDA-induced molecular events, without altering intracellular Ca^2+^ levels. Therefore, it is likely that K9-C-peptide attenuates glutamate excitotoxicity through the suppression of oxidative stress and its downstream pathways.

Proinsulin C-peptide is now recognized as a bioactive peptide that protects against both microvascular and macrovascular complications associated with type 1 diabetes, including diabetic retinopathy, neuropathy, and cardiovascular disease [20,24,37]. In vascular smooth muscle, C-peptide exerts anti-inflammatory and anti-atherogenic effects in type 1 and early type 2 diabetes [21]. However, the clinical use of C-peptide has been constrained by its relatively short plasma half-life of approximately 30 min, underscoring the need for the development of sustained delivery strategies [24]. To overcome this pharmacokinetic limitation, human C-peptide has been recombinantly fused with a thermosensitive lysine-containing ELP, and the resulting K9-C-peptide allows sustained release of human C-peptide into the circulation for up to 19 days or into the ocular space for up to 8 weeks [24,31].

This prolonged C-peptide delivery platform enables the investigation of the therapeutic effects of human C-peptide against diabetic aortic dysfunction by systemic delivery and against hyperglycemia-induced retinal neovascularization by intraocular delivery, thereby eliminating the need for frequent administration [24,31]. In this study, K9-C-peptide conferred sustained protection for at least three weeks against NMDA-induced RGC loss and retinal thinning, as well as axonal transport impairment. Notably, an equimolar dose of native human C-peptide alone failed to provide comparable protection, highlighting the importance of sustained bioavailability for preventive efficacy, rather than suggesting that native human C-peptide is inherently ineffective. Considering the chronic and progressive course of retinal degenerative diseases, sustained-release platforms such as K9-C-peptide may provide a clinically translatable approach for the treatment of diabetic complications and other vision-threatening retinal disorders, including glutamate excitotoxicity.

Experimental studies targeting glutamate excitotoxicity have focused on the inhibition of oxidative stress, inflammation, and reactive gliosis in glutamate excitotoxicity animal models [2,8,14,15]. In NMDA-induced murine models, NMDA-induced RGC damage and inner retinal thinning are reduced by tert-butylhydroquinone, and these protective effects are associated with activation of the Nrf2 signaling pathway and inhibition of oxidative stress and inflammation [38,39]. Hesperidin, a plant-derived bioflavonoid, exerts a neuroprotective effect against NMDA-induced retinal degeneration by attenuating oxidative stress, excessive calpain activation, and neuroinflammation [4,8]. Necrostatin-1 and GSK872, inhibitors of receptor-interacting protein 1 and 3, respectively, prevent NMDA-induced RGC loss and retinal damage, which is associated with the inhibition of the Nod-like receptor family, pyrin domain containing 3 (NLRP3) inflammasome [2]. Moreover, in kainic acid-induced excitotoxicity mouse models, withaferin A, a plant steroidal lactone, attenuates TNF-α-mediated neuronal apoptosis through inhibition of reactive gliosis [15]. Despite a number of therapeutic candidates demonstrating therapeutic potential for glutamate excitotoxicity-associated retinal degeneration in animal models, these potential candidates still require additional preclinical and clinical studies to fully establish their effectiveness, safety, and optimal administration regimens.

Taken together, our findings suggest that sustained modulation of downstream pathological events may be critical for effective neuroprotection in glutamate excitotoxicity. Although excessive activation of NMDA receptors initiates retinal injury, targeting oxidative stress, mitochondrial dysfunction, and inflammation appears to provide more durable protective effects. In this context, K9-C-peptide attenuated multiple NMDA-induced pathological processes while maintaining prolonged intraocular bioavailability. These findings indicate that K9-C-peptide exerts neuroprotective effects through the coordinated regulation of key downstream pathways involved in RGC degeneration. In addition, the preservation of axonal transport function by K9-C-peptide suggests its potential to prevent early neuronal dysfunction associated with glaucomatous neurodegeneration. Given these properties, K9-C-peptide may represent a promising long-acting interventional strategy for the treatment of glutamate excitotoxicity and other retinal neurodegenerative diseases.

Several limitations of this study should be acknowledged. First, our findings are based on an NMDA-induced mouse model, that, while widely used, does not completely reflect the chronic, progressive, and multifactorial pathophysiology of human glaucoma. Therefore, validation in additional preclinical models, as well as in human retinal tissues and optic nerves, will be necessary to strengthen the translational relevance. Second, the precise signaling intermediates linking human C-peptide to the suppression of NMDA-induced molecular events remain to be elucidated. In particular, future studies exploring receptor-mediated signaling pathways will provide deeper mechanistic insight. Third, the improvement in CTB transport may be secondary to increased RGC survival rather than a direct protective effect on axonal transport. Therefore, future studies examining the direct effect of K9-C-peptide on axonal transport would further strengthen its translational relevance. Finally, further studies are warranted to optimize dosing and administration regimens, evaluate long-term efficacy, and assess potential therapeutic benefits in combination with IOP-lowering agents for clinical applications.

## 5. Conclusions

In conclusion, prolonged intraocular delivery of human C-peptide using K9-C-peptide confers durable protection against NMDA-induced retinal degeneration and optic nerve axonal transport impairment. These protective effects were associated with suppression of NMDA-induced oxidative stress, mitochondrial dysfunction, reactive gliosis, and inflammation, without altering intracellular Ca^2+^ levels. These findings establish K9-C-peptide as a long-acting protective candidate for the prevention of RGC death and optic nerve axonal transport deficits, and provide insight into the potential mechanisms underlying C-peptide-based interventions in retinal glutamate excitotoxicity.

## Data Availability

All data associated with this study are present in the paper. The raw data are available from the corresponding author upon reasonable request.

## Supplementary Materials

The following supporting information can be downloaded at: https://www.mdpi.com/article/10.3390/antiox15070911/s1, Figure S1: Sustained intraocular delivery of K9-C-peptide.

## Abbreviations

ANOVA	one-way analysis of variance
Brn3a	brain-specific homeobox/POU domain protein 3A
CTB	cholera toxin subunit B
DHE	dihydroethidium
GFAP	glial fibrillary acidic protein
GLAST	glutamate-aspartate transporter
H&E	hematoxylin and eosin
H-BAPTA	hydrogel-formulated BAPTA
H-NAC	hydrogel-formulated NAC
H-Trolox	hydrogel-formulated Trolox
Iba-1	ionized calcium-binding adaptor molecule 1
IOP	intraocular pressure
IL-6	interleukin-6
IRL	inner retinal layer
MDA	malondialdehyde
NAC	N-acetyl-L-cysteine
NHS	N-hydroxysuccinimide
NMDA	N-methyl-D-aspartate
OCT	optical coherence tomography
RGC	retinal ganglion cell
ROS	reactive oxygen species
∆Ψm	mitochondrial membrane potential
TNF-α	tumor necrosis factor-α
