# Supplementary material for: Biopolymer-Conjugated Human C-Peptide Provides Sustained Neuroprotection and Preserves Axonal Transport in a Mouse Model of NMDA-Induced Retinal Degeneration via Antioxidative Mechanisms
Source: Antioxidants (Basel). 2026 Jul 22;15(7):911. doi: 10.3390/antiox15070911 (PMC13403778; doi:10.3390/antiox15070911)

Supplementary Figure 1. Sustained intraocular delivery of K9-C-peptide. C57BL/6 mice were intravitreally injected with fluorescein-conjugated human C-peptide (1.2 μg) or K9-C-pep (20 μg). Intraocular fluorescence images were obtained for 21 days post-injection. Scale bar, 300 μm.

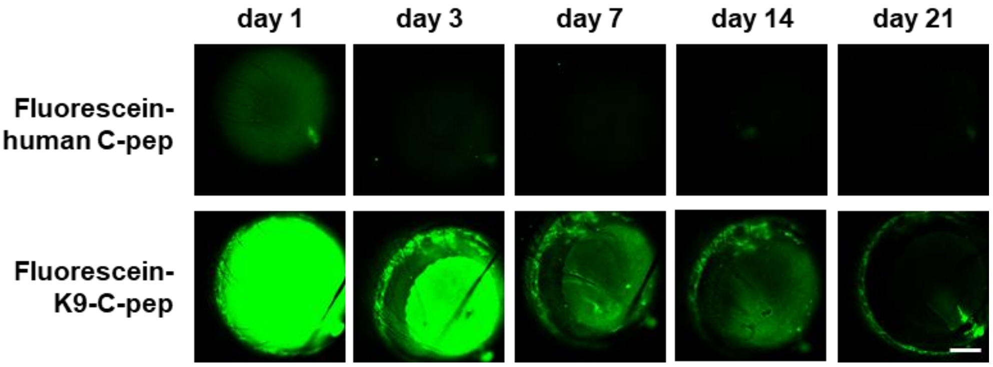

Supplement: Supplementary file 1 [file antioxidants-15-00911-s001.zip › antioxidants-4408338-supplementary.pdf]
